# Residual LSTM-based short duration forecasting of polarization current for effective assessment of transformers insulation

**DOI:** 10.1038/s41598-023-50641-z

**Published:** 2024-01-16

**Authors:** Aniket Vatsa, Ananda Shankar Hati, Prashant Kumar, Martin Margala, Prasun Chakrabarti

**Affiliations:** 1 Department of Electrical Engineering, Indian Institute of Technology (Indian School of Mines), Dhanbad, 826004 India; 2 Department of Mechanical, Robotics, and Energy Engineering, Dongguk University-Seoul, 30 Pil-dong 1 gil, Jung-gu, Seoul, 04620 Republic of Korea; 3 School of Computing and lnformatics, University of Louisiana at Lafayette, Lafayette , USA; 4 Department of Computer Science and Engineering, Sir Padampat Singhania University, Udaipur, Rajasthan 313601 India

**Keywords:** Engineering, Electrical and electronic engineering

## Abstract

The empirical application of polarization and depolarization current (PDC) measurement of transformers facilitates the extraction of critical insulation-sensitive parameters. This technique, rooted in time-domain dielectric response analysis, forms the bedrock for parameterization and insulation modeling. However, the inherently time-consuming nature of polarization current measurements renders them susceptible to data corruption. This article explores deep-learning-based short-duration techniques for forecasting polarization current to address this limitation. By incorporating spatial shortcuts, the residual long short-term memory (LSTM) network facilitates the seamless propagation of spatial and temporal gradients. Furthermore, the relative forecasting assessment of the proposed residual LSTM model’s performance is made against traditional LSTM, attention LSTM, gated recurrent units (GRU), and convolutional neural network (CNN) models. Thus, optimal model selection strategies are evaluated based on their capability to capture extended dependencies and short-term information present in the data. In addition, the Monte Carlo dropout prediction is employed to estimate uncertainty in polarization current forecasts. The findings demonstrate that the proposed residual LSTM network model for polarization current forecasting yields the lowest error metrics and maintains prediction consistency over the testing duration. Thus, the proposed approach significantly reduces PDC measurement time, providing an effective means to develop proactive maintenance strategies for evaluating the insulation condition of transformers.

## Introduction

Ensuring the integrity of transformer insulation stands as an imperative facet for the seamless functioning of power system networks^1^. Transformer insulation is subjected to multiple stress factors during its operational life, leading to the deterioration of oil-paper insulation^2^. Therefore, effective monitoring and assessment of insulation conditions are essential to mitigate the risk of catastrophic failure and subsequent power outages^3^. In that regard, chemical-based methodologies, such as dissolved gas analysis (DGA), furan analysis, and degree of polymerization (DP), are employed to discern the condition of transformer insulation, albeit their application is constrained in assessing insulation integrity comprehensively^4^. Furthermore, DGA analysis is susceptible to the influence of gas fluctuations, thereby potentially impeding the accuracy of results. Moreover, DP measurements entail a destructive nature, imposing constraints on the frequency and extent of testing, and the diagnostic scope of furan analysis is inherently limited, addressing specific aspects of transformer insulation health. On the other hand, the utilization of dielectric response analysis methodologies, such as polarization and depolarization current (PDC) and recovery voltage measurement (RVM), has experienced remarkable proliferation in recent years due to their non-invasive nature and highly efficient dynamic insulation characterization capabilities^5^. By critically examining PDC data, valuable insights into age-sensitive parameters that influence the operational health of a transformer can be derived^6^.

The literature emphasizes the importance of time domain analysis techniques, such as PDC analysis concerning transformer oil-paper insulation, serving as a pivotal aspect of effective insulation assessment^7^. Diverse traditional insulation modeling techniques encompassing the conventional Debye mode (CDM) and the modified Debye model (MDM) provide a unique characterization of transformer insulation systems^8,9^. Furthermore, advanced techniques such as the model with time-varying parameters (MTVP) and the modified Maxwell model (MMM) provide a foundation for sophisticated methodologies to comprehend and evaluate insulation performance^10^. Furthermore, the insulation-sensitive parameters extracted from PDC analysis, such as DC conductivity, transfer function zero, and detrapped charge, play a decisive role in predictive analysis, facilitating prognostication of insulation conditions of transformers^11,12^. The application of PDC analysis and its associated insulation modeling techniques augments transformer insulation systems’ comprehension and prognostic capabilities^13^. Nevertheless, an aspect warranting attention is the discernible possibility of the PDC technique introducing a noteworthy extension to measurement times, occasionally encompassing durations of several hours. Consequently, this prolonged duration renders the acquired low-magnitude PDC data vulnerable to potential corruption stemming from the deleterious effects of noise and adverse environmental conditions. Therefore, an escalating demand exists for novel and time-efficient PDC measurement methodologies. Although traditional models like autoregressive integrated moving average (ARIMA), error-trend-seasonality (ETS), and neural networks (NN) have been proposed for predicting polarization current^14^, they grapple with challenges in terms of outlier sensitivity and effectively handling seasonality and trends. In recognition of these limitations, researchers are actively exploring alternative avenues, such as leveraging deep learning techniques, to enhance transformer performance parameter estimation. The key novelties and contributions of the investigation can be given as follows:

1. A deep learning-based forecasting method based on residual long short-term memory (LSTM) networks is proposed in this article for short-duration PDC measurement of transformers’ insulation systems.
2. The optimal polarization current forecasting model is established by relative forecasting assessment of the proposed residual LSTM model with attention-LSTM, LSTM, gated recurrent units (GRU), and convolutional neural network (CNN) models.
3. The Monte Carlo dropout prediction approach, is utilized to enhance uncertainty estimation, contributing to polarization forecasts by estimating the mean and standard deviation of predictions across Monte Carlo samples to quantify forecast confidence.
4. In order to ensure the robustness of the proposed residual LSTM model, its forecasting capabilities in two distinct scenarios, each employing a different historical data window for prediction, are validated.
5. A crucial insulation-sensitive parameter, the DC conductivity of the transformer oil-paper insulation system, is determined based on the predicted polarization current for expedient insulation condition assessment.

The rest of the article is structured as follows. The “Brief theory of the PDC approach ” section introduces the PDC approach for analyzing polarization current in transformer insulation, detailing its theoretical foundations based on dipole alignment and dielectric response functions. The “Proposed short-duration PDC forecasting methodology” section details the PDC forecasting methodology, covering sample preparation and residual LSTM architecture to enhance temporal pattern identification, emphasizing gradient flow improvement. The “Experimental results” section presents the results for a deep learning residual LSTM forecasting model trained under two distinct temporal contexts and also comparatively analyses the residual LSTM model’s performance. Additionally, uncertainty analysis is conducted using a Monte Carlo dropout prediction method. The “Discussion” section draws crucial insights from the results. Eventually, the “Conclusion” section concludes the investigation.

## Brief theory of the PDC approach

The polarization current in transformer insulation, which is crucial for evaluating such complex insulation systems, originates from alignment dipoles with applied electric fields to create displacement current^15^. In an oil-paper insulation (OPI) system, interfacial and electronic polarization are prominent, and it is often followed by distinct relaxation of dipole groups after the electric field is removed. This relaxation behavior can be represented using RC branches with different time constants. The current density $j(t)$ generated by dielectric material under the influence of an external electric field $E(t)$ while considering the macroscopic polarization $P(t)$ effect can be represented as^16^:

1 $$\begin{aligned} j(t)=\sigma _{o}E(t)+\varepsilon _{o} \frac{ dE(t)}{dt}+\frac{ dP(t)}{ dt} \end{aligned}$$

In this context, the symbols $\sigma _{o}$ and $\varepsilon _{o}$ represent the DC conductivity and vacuum permittivity, respectively. Polarization can be calculated from the convolution in the time domain of the dielectric response function *f*(*t*) and electric susceptibility $\chi$.

2 $$\begin{aligned} P(t)=\varepsilon _{o}\chi E(t)+ \varepsilon _{o} \int _{o}^{t} f(t-\tau ) E(\tau ) d\tau \end{aligned}$$

The equation describes material polarization in response to an electric field where the first term captures immediate polarization, determined by electric susceptibility and applied field; the second term involves convolution of the dielectric response function and field history, characterizing cumulative polarization due to delayed responses and relaxation processes. By utilizing Eqs. (1) and (2), the current density can be further modified as follows:

3 $$\begin{aligned} j(t)=\sigma _{o}E(t)+\varepsilon _{o}(1+\chi ) \frac{\delta E(t)}{dt}+ \varepsilon _{o} \frac{d}{dt} \int _{o}^{t} f(t-\tau ) E(\tau ) d\tau \end{aligned}$$

This equation can be subsequently simplified to deduce the polarization current. Furthermore, for a direct current test voltage $U_{o}$, the polarization current ($i_{pol}$) is intricately influenced by essential factors such as the geometric capacitance ($C_{o}$), the electric field ($E$), the direct current conductivity ($\sigma _{o}$), the vacuum permittivity ($\varepsilon _{o}$), the transient polarization component ($\delta (t)$) which is neglected, and the dielectric response function ($f(t)$) as follows:

4 $$\begin{aligned} i_{pol}=C_{o}U_{0}[\frac{\sigma _{o}}{\varepsilon _{o}}+f(t)] \end{aligned}$$

The foundation of transformer insulation models rests upon utilizing dielectric spectroscopy-based PDC measurements. This approach facilitates the extraction of a wide array of parameters sensitive to the insulation properties. Furthermore, the developed models can capture intricate details concerning the insulation’s behavior and performance, enabling a comprehensive understanding of the transformer’s health and efficiency.

**Figure 1:**
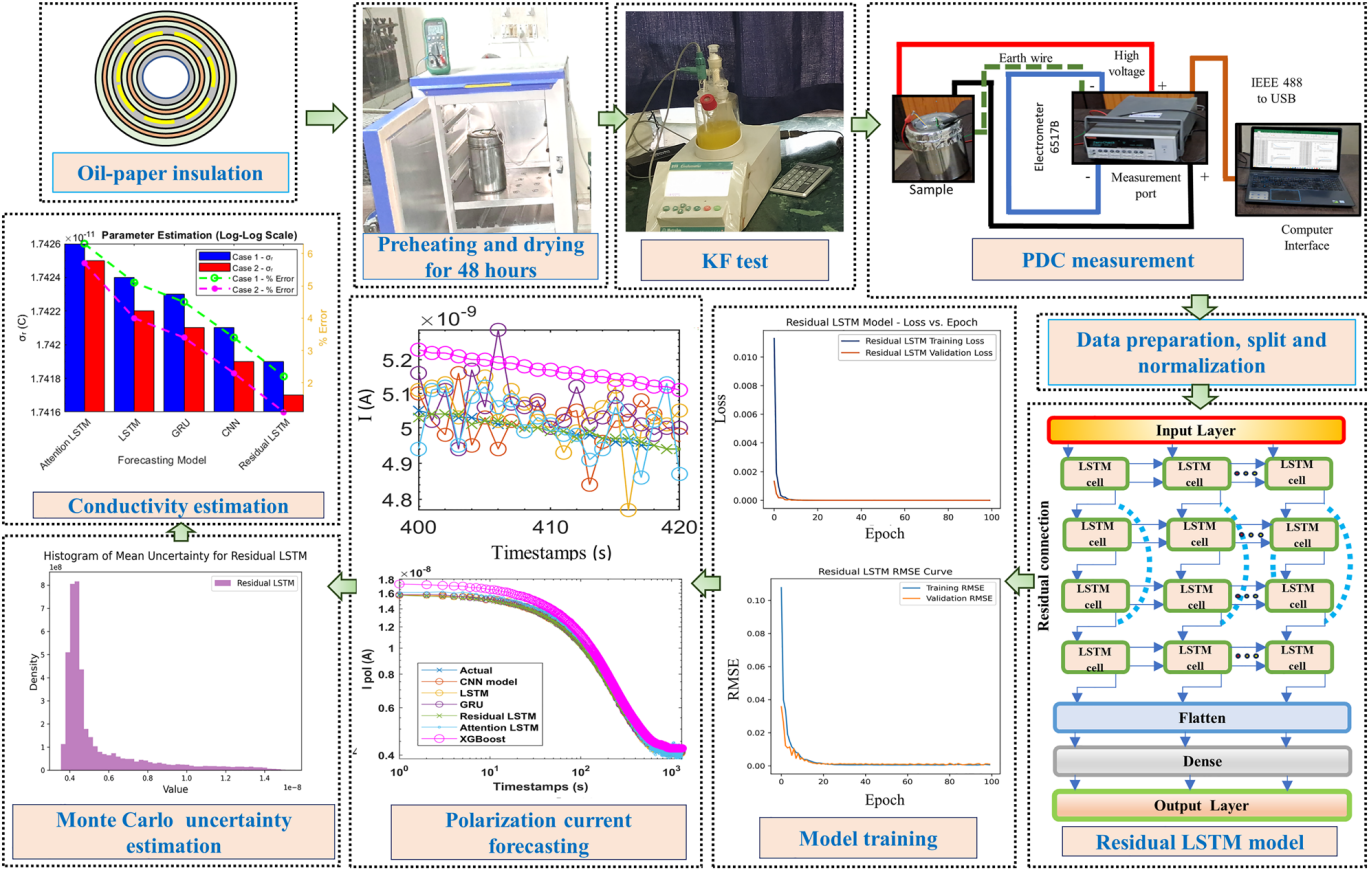
The topological configuration for efficient short-duration polarization current forecasting framework using residual LSTM network.

## Proposed short-duration PDC forecasting methodology

The extended measurement duration of low-magnitude PDC signals, which can last for several hours, presents a substantial measurement challenge. It leaves the acquired data susceptible to potential corruption from noise and environmental conditions. Therefore, there is a growing interest in efficient short-duration PDC measurement methodologies. Deep learning techniques hold the potential to address this challenge by enhancing the measurement process, leading to shorter measurement times and ultimately elevating the precision and dependability of polarization current forecasts. This approach addresses the underlying problem by introducing a more efficient and reliable means of obtaining PDC measurements. The comprehensive polarization current forecasting scheme using the residual LSTM network is shown in Fig. 1.

**Figure 2:**
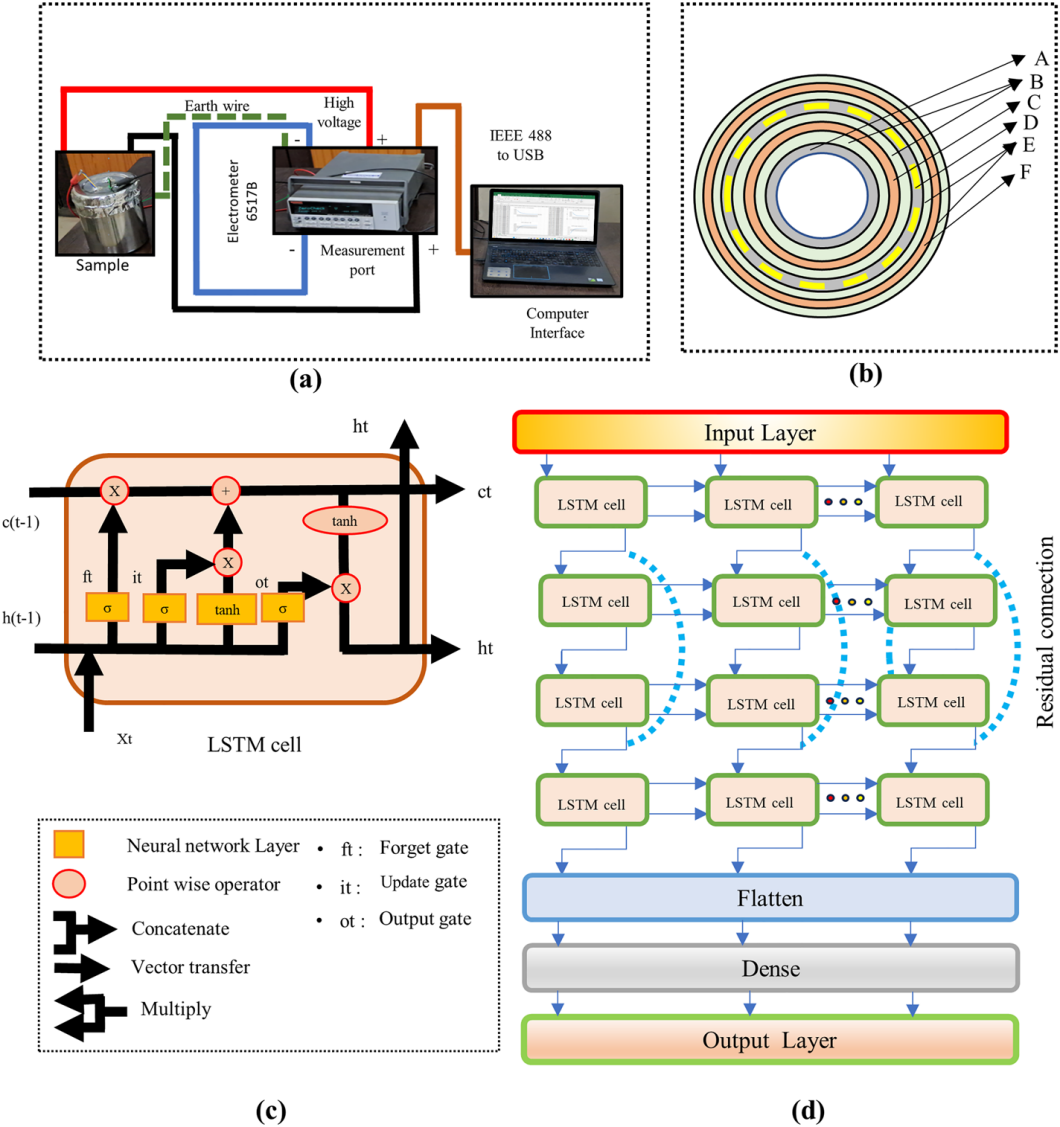
(**a**) Diagram depicting the polarization current measurement procedure, (**b**) Construction of the OPI test sample, (**c**) Schematics of an LSTM cell displaying gating variables, and (**d**) Architecture of the proposed residual LSTM model.

The preparation of the insulation specimens involved arranging layers comprising a pressboard cylinder (A), kraft paper (B, E), and high and low-voltage transformer windings (C, F) utilizing copper foil. Furthermore, pressboard strips are used to emulate oil ducts (D). Moreover, this approach enables a comprehensive study of polarization phenomena within a controlled laboratory environment. In this study, the samples underwent initial treatment to remove pre-absorbed moisture by subjecting them to heating at 90°. Afterward, the insulation sample is impregnated in mineral oil to simulate operational conditions, ensuring an accurate representation of the composite insulation system of transformers. Post-treatment, a Karl Fischer (KF) titration method was used to determine moisture content subsequently after allowing sufficient time for attaining equilibrium. In order to investigate the insulation analysis of the oil-paper insulation system, the polarization current is measured using an electrometer technique. Figure 2a illustrates the PDC measurement approach employed for the OPI sample, utilizing a direct current (DC) excitation voltage of 1000 V. Furthermore, Fig. 2b presents an overhead schematic view of the constructed OPI sample. A residual LSTM-based deep learning forecasting methodology is then utilized for the investigation of reducing the cumulative PDC measurement time by effectively forecasting polarization current.

The Residual LSTM approach extends the conventional LSTM architecture (depicted in Fig. 2c) by introducing residual connections, enabling it to capture long-range dependencies and complex temporal patterns concealed within the sequential data^17^. These connections help mitigate the vanishing and exploding gradient problem, leading to improved learning and forecasting accuracy of polarization current forecasting problem. The residual LSTM model architecture proposed in this study involves multiple LSTM layers, as shown in Fig. 2d, with each layer comprising 64 units configured to return sequences.

An input layer precedes a single, 64-unit LSTM layer in the attention-based LSTM model, which is then enhanced by a unique attention mechanism. A tanh-activated dense layer, permutation layers for dimension reshaping, and a softmax-activated dense layer constitute the attention mechanism. These components function together to highlight crucial phases in the input sequence. The fundamental LSTM model takes a more simplified approach, with a single 64-unit LSTM layer and an output layer. The GRU model, a highly effective LSTM variant, consists of an input layer, a GRU layer with 64 units, and an output layer. In contrast, the proposed polarization current forecasting model incorporates a multi-layered LSTM structure, with each layer comprising 64 neurons. A notable feature is establishing a sequential flow of information, where the first LSTM layer returns sequences to the second, facilitating a coherent exchange of information. The distinctive aspect of this architecture is the incorporation of residual connections, a pivotal mechanism that facilitates seamless information transfer between the LSTM layers. By effectively capturing intricate temporal patterns and long-term dependencies in the data, the model becomes adept at complex sequence modeling tasks such as polarization current forecasting. Following the series of LSTM layers, a flattened layer is employed to reshape the output into a compact 1D vector. This output is then fed into a dense layer, which is responsible for generating predictions for the forecasted time steps as defined by the specified forecast horizon. The residual LSTM introduces spatial shortcuts, thereby enabling the smooth flow of both spatial and temporal gradients^18^. The strategic integration of the residual LSTM approach contributes significantly to the model’s ability to surmount challenges posed by gradient-related issues, ultimately enhancing its forecasting accuracy and making it a promising solution for capturing complex temporal dynamics in various applications. To comprehensively evaluate the forecasting capabilities of the residual LSTM model, two distinct cases are investigated in this article. Specifically, the cases involve employing different lookback periods: one instance with a lookback of 60 (referred to as case 1) and another instance with a lookback of 100 (referred to as case 2). This approach allows for a thorough examination of the model’s performance under varying lookback settings, thereby providing valuable insights into its effectiveness across different temporal contexts. The study employed various hyperparameters in the deep learning model, including the number of LSTM layers, learning rate, and sequence length, to predict the insulation condition of a transformer through polarization current forecasting. The combination of grid search and manual fine-tuning method was employed to ensure the selection of the best hyperparameters.

In a residual LSTM model, an auxiliary spatial shortcut connection is introduced in a traditional LSTM architecture to model the temporal dependencies effectively. Thereby, it facilitates proper gradient flow and mitigates the vanishing gradient problem. Furthermore, at each time step $t$, the input $\textbf{x}_t$ is processed, and the output hidden state $\textbf{h}_t$ is computed. Simultaneously, a series of gating mechanisms update the memory cell state $\textbf{c}_t$. These gating variables are computed using various characteristic equations, as represented below:

5 $$\begin{aligned} i_t= & {} \sigma (W_{xi} \textbf{x}t + W{hi} \textbf{h}_{t-1} + \textbf{b}_i) \end{aligned}$$

6 $$\begin{aligned} f_t= & {} \sigma (W_{xf} \textbf{x}t + W{hf} \textbf{h}_{t-1} + \textbf{b}_f) \end{aligned}$$

7 $$\begin{aligned} o_t= & {} \sigma (W_{xo} \textbf{x}t + W{ho} \textbf{h}_{t-1} + \textbf{b}_o) \end{aligned}$$

8 $$\begin{aligned} \tilde{\textbf{c}}t= & {} \tanh (W{xc} \textbf{x}t + W{hc} \textbf{h}_{t-1} + \textbf{b}_c) \end{aligned}$$

9 $$\begin{aligned} \textbf{c}t= & {} f_t \odot \textbf{c}{t-1} + i_t \odot \tilde{\textbf{c}}_t \end{aligned}$$

10 $$\begin{aligned} \textbf{h}_t= & {} o_t \odot \tanh (\textbf{c}_t) \end{aligned}$$

Here, $\sigma$ denotes the sigmoid activation function, $W$ matrices, and $\textbf{b}$ vectors represent the learnable weights and biases associated with input and hidden states. Furthermore, $\odot$ indicates element-wise multiplication. The residual connection is introduced by adding the output $\textbf{h}_t$ to the input $\textbf{x}_t$^19^, creating a shortcut trail that facilitates learning incremental insights by leveraging the gradient information expressed as:

11 $$\begin{aligned} \text {residual} = \textbf{h}_t + \textbf{x}_t \end{aligned}$$

This mechanism enables addressing the vanishing gradient problem and enhances the capacity of the network to capture both short-term and long-term patterns in the data.

## Experimental results

This research paper analyzes a deep learning residual LSTM forecasting model’s performance when trained under two distinct temporal contexts of different past timestamps under case 1 and case 2. The lookback parameter determines the number of past time steps considered input for the LSTM model, impacting its ability to capture temporal dependencies and patterns within the data. Furthermore, to select the optimal forecasting model, a relative comparison of the proposed residual LSTM model is also made with similar approaches such as attention LSTM, LSTM, GRU and CNN models. To assess the model’s accuracy and generalization across various temporal contexts, essential metrics such as mean squared error (MSE), mean absolute error (MAE), and root mean squared error (RMSE) are employed for performance evaluation.

The MSE quantifies the average squared difference between the actual polarization current value ($i_{\textrm{pol}}$) and the predicted polarization current value ($i_{\textrm{pol}}^{for}$). The objective of the training process is to minimize the loss function $\Psi$ by optimizing the model’s trainable parameters, such as weights and biases. This optimization is achieved through the Adam optimizer, a widely utilized stochastic gradient descent (SGD) variant. It employs adaptive learning rates, momentum, and second-moment estimates of the gradients to navigate the loss landscape efficiently. The iterative update of model parameters concerning the loss function aims to converge towards the global minima. This results in a model that can provide more precise and accurate forecasts as it learns to capture patterns and relationships within the data. The loss function between the forecasted and original polarization current is computed as follows:

12 $$\begin{aligned} \mathrm {\Psi _{Loss~mse}} = \frac{1}{N} \sum _{i=1}^{N} \left( i_{\textrm{pol}} - i_{\textrm{pol}}^{for}\right) ^2 \end{aligned}$$

Where $N$ represents the number of instances, $i_{\textrm{pol}}$ is the actual polarization current value, and $i_{\textrm{pol}}^{for}$ is the predicted polarization current value. After training is commenced, MSE, MAE, and RMSE performance metrics are used for the evaluation of the proposed forecasting model on the test data set. Similarly, the mean absolute error measures the average absolute difference between the actual ($i_{\textrm{pol}}$) and predicted ($i_{\textrm{pol}}^{for}$) polarization current values are evaluated as follows :

13 $$\begin{aligned} \textrm{MAE} = \frac{1}{N} \sum _{i=1}^{N} \left| i_{\textrm{pol}} - i_{\textrm{pol}}^{for}\right| \end{aligned}$$

Where $N$ represents the number of instances, $i_{\textrm{pol}}$ is the actual polarization current value, and $i_{\textrm{pol}}^{for}$ is the predicted polarization current value. Additionally, the root mean squared error provides a square root of the average squared differences between the actual ($i_{\textrm{pol}}$) and predicted ($i_{\textrm{pol}}^{for}$) polarization current values, offering a more balanced representation of errors. It can be defined as:

14 $$\begin{aligned} \textrm{RMSE} = \sqrt{\frac{1}{N} \sum _{i=1}^{N} \left( i_{\textrm{pol}} - i_{\textrm{pol}}^{for}\right) ^2} \end{aligned}$$

Where $N$ represents the number of instances, $i_{\textrm{pol}}$ is the actual polarization current value, and $i_{\textrm{pol}}^{for}$ is the predicted polarization current value.

**Figure 3:**
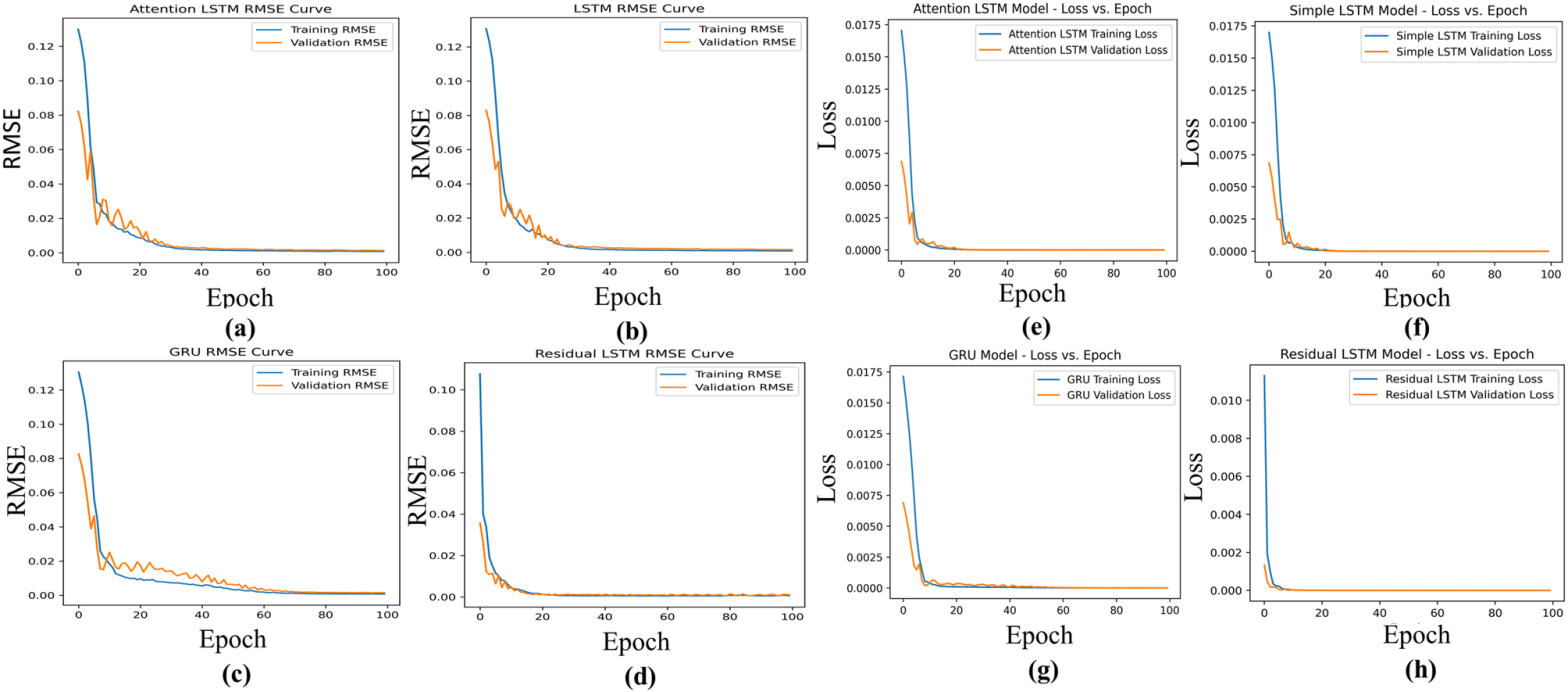
RMSE curves for (**a**) Attention LSTM, (**b**) LSTM, (**c**) GRU, and (**d**) Residual LSTM models; Loss curves for (**e**) Attention LSTM, (**f**) LSTM, (**g**) GRU, and (**h**) Residual LSTM models.

**Figure 4:**
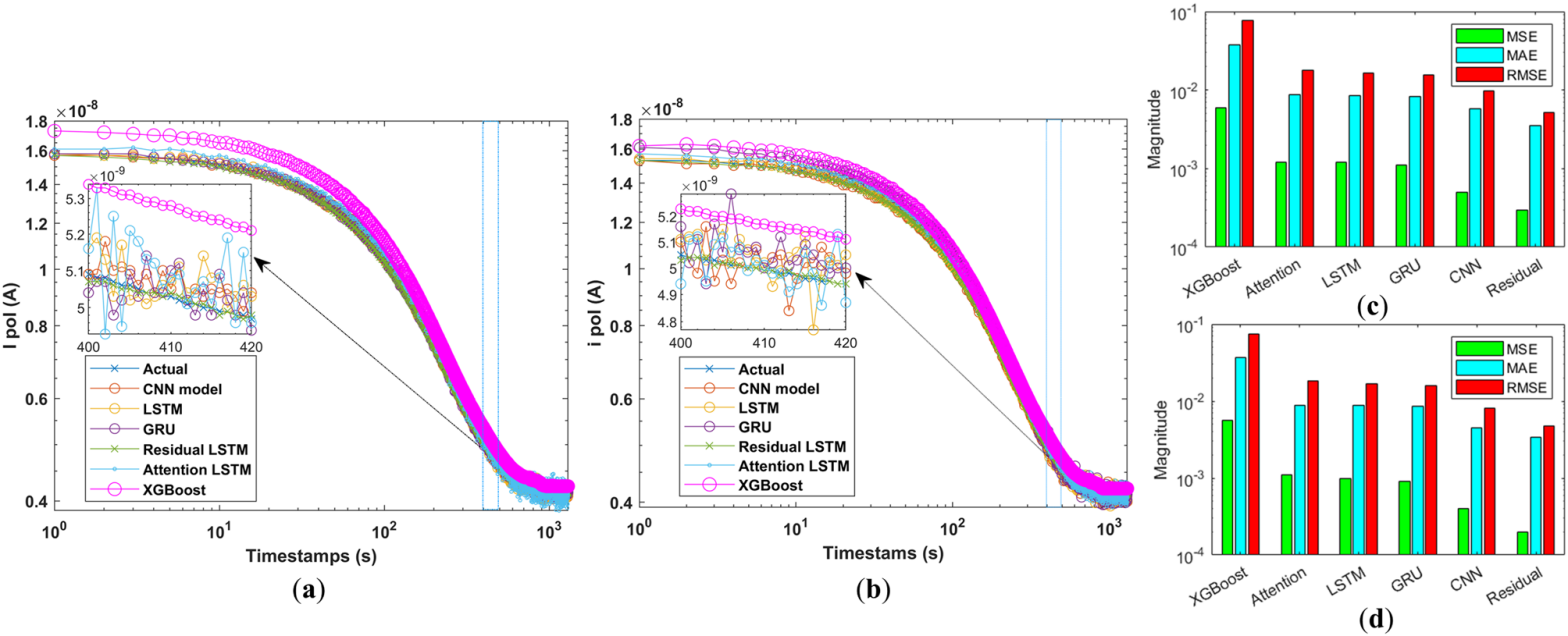
Comparison of polarization current forecasting performance of different models for (**a**) Case 1 and (**b**) Case 2; Polarization current forecasting metrics (**c**) Case 1 and (**d**) Case 2.

Figure 3a–d illustrates the RMSE curve of the trained forecasting model across epochs. It becomes apparent that the initial transition in validation RMSE swiftly converges after 20 epochs for the residual LSTM model. In contrast, for the attention of LSTM, LSTM, and GRU models, a prolonged transition is distinctly observable, suggesting that the proposed forecasting model exhibits more efficient convergence dynamics. Figure 3e–h illustrates the progression of the loss curve across epochs of the different forecasting models. Notably, it becomes evident that the initial transition in validation loss swiftly converges within the first ten epochs for the residual LSTM model. In contrast, a more prolonged transition and higher loss are distinctly observable in the attention LSTM, LSTM, and GRU models. This distinction underscores the superior convergence dynamics exhibited by the proposed forecasting model. The forecasting performance of attention LSTM, LSTM, GRU, CNN and residual LSTM models in the test set are shown in Fig. 4. Additionally, the models’ forecasting performance is examined under two distinct cases: Case 1 and Case 2, as depicted in Fig. 4a and b, respectively. It becomes evident that attention LSTM, LSTM, and GRU models deliver elevated initial predictions. Particularly, the attention LSTM model demonstrates increased oscillations at the forecast horizon, while the CNN models showed improved initial forecasting. In contrast, the residual LSTM not only maintains a low initial forecasting error but also showcases minimal oscillations. This observation underscores the advantage of the residual LSTM model in achieving both accurate initial predictions and stable forecasting outcomes.

**Table 1:** Average polarization current forecasting metrics of different approaches in the test set.

Models	Case 1			Case 2		
	MSE	MAE	RMSE	MSE	MAE	RMSE
XGBoost	0.0059	0.0377	0.0766	0.0056	0.0370	0.0751
Attention	0.0012	0.0087	0.0181	0.0011	0.0089	0.0184
LSTM	0.0012	0.0085	0.0164	0.0010	0.0087	0.0166
GRU	0.0011	0.0082	0.0154	0.0009	0.0086	0.0160
CNN	0.0005	0.0058	0.0098	0.0004	0.0045	0.0081
Residual	0.0003	0.0035	0.0052	0.0002	0.0034	0.0048

Table 1 presents the polarization current forecasting results of different predictive models using two different lookback values: 60 and 100. The plot in Fig. 4c,d compares the MSE, MAE, and RMSE values for each model and lookback period, highlighting their respective forecasting accuracies. It is important to note that the XGBoost model’s performance remains unaffected by the variation in lookback, aligning with its inherent characteristic of not directly considering temporal dependencies. Among the sequence-based models, the residual LSTM stands out as the superior performer with both lookback settings, exhibiting the lowest MSE, MAE, and RMSE values. This superiority can be attributed to the residual LSTM’s architecture, incorporating residual connections to capture intricate long-term patterns within the data effectively. On the other hand, the attention LSTM, LSTM, GRU and CNN models exhibit comparable but slightly higher errors, which can be detrimental for such low-magnitude signals. However, the XGBoost model shows the highest errors among all approaches, indicating that for this specific polarization current forecasting task, the residual LSTM model emerges as the preferred choice due to its robust predictive capability and ability to capture complex temporal dependencies effectively.

**Figure 5:**
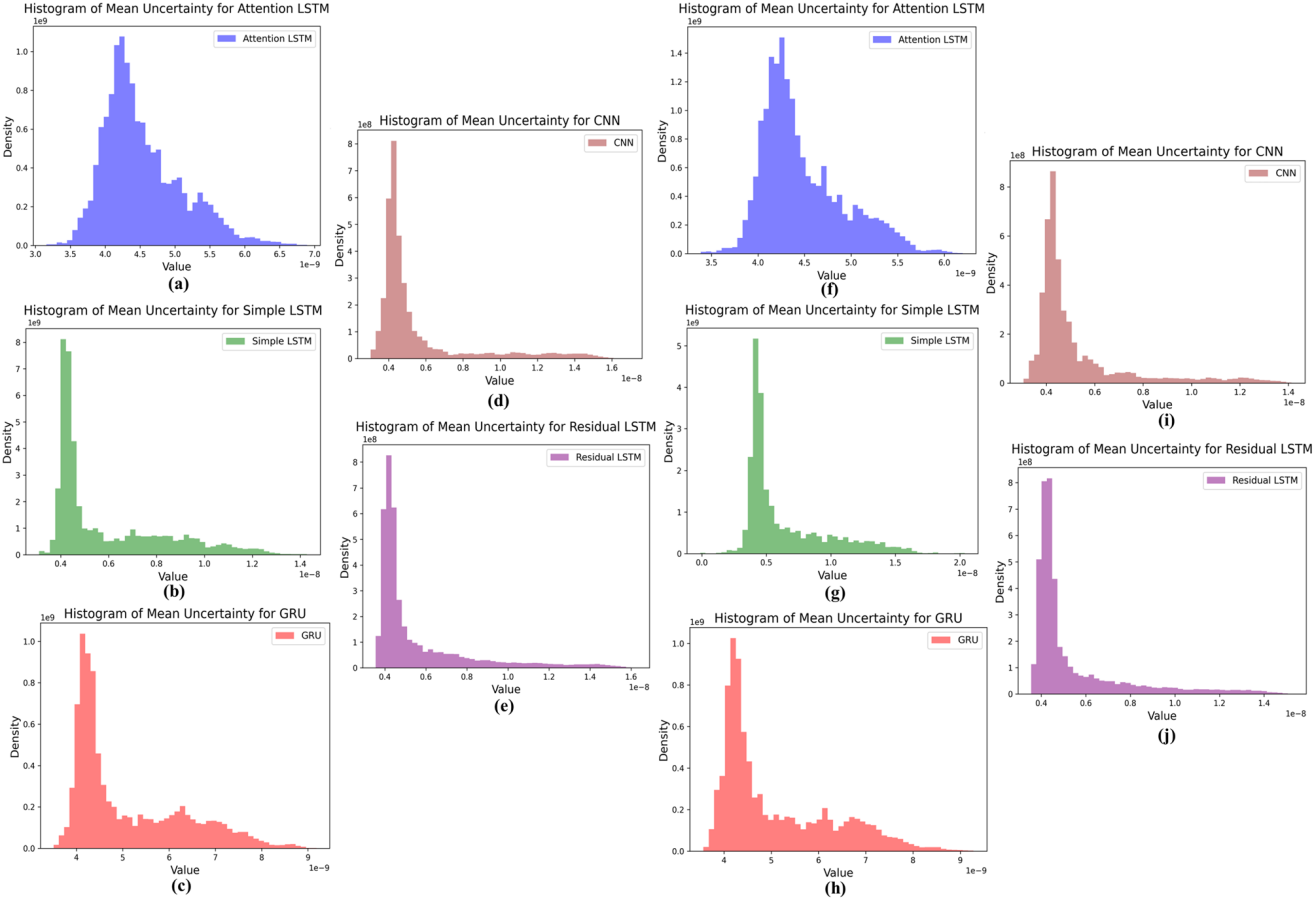
Histograms depicting the mean uncertainty of various polarization current forecasting models. Figures (**a**–**e**) are presented for Case 1, and Figures (**f**–**i**) for Case 2.

A Monte Carlo dropout prediction method, is implemented in this research paper to estimate the uncertainty of the polarization current forecasting models. In this context, for each deep learning model, the Monte Carlo dropout prediction function conducts a specified number of forward passes. These forward passes of Monte Carlo samples traverse the model and gather corresponding predictions^20,21^. The mean and standard deviation of these predictions across the number of Monte Carlo samples serve as crucial indicators of uncertainty, reflecting the model’s inherent variability and quantifying prediction confidence in forecasting outcomes. In the context of Monte Carlo uncertainty estimation, multiple predictions are generated by applying dropout during the testing phase. Each prediction is a sample from the distribution of possible outcomes given the model’s uncertainty. Each model’s Monte Carlo dropout predictions result in a set of mean uncertainty values. The histograms visualize the distribution of mean uncertainties for each model by representing the probability density, which is a normalized measure of the likelihood of a particular range of mean uncertainty values. The analysis of Fig. 5a–j reveals the uncertainty associated with different models. Specifically, the residual LSTM model exhibits the most favorable outcome, manifesting the lowest uncertainty values. This achievement is underscored by the model’s low peak density, which materializes around $0.4\times 10^{-8}$, achieved through the utilization of the preceding 60 seconds of historical data for predictive purposes. In contrast, the simple LSTM model displays the highest peak density level, reaching up to $8\times 10^{9}$, while the attention LSTM model follows with a peak density of approximately $1.75\times 10^{9}$. Notably, the CNN model exhibits a reduction in uncertainty; however, its uncertainty in later stages remains higher than that of the residual LSTM model. This discrepancy becomes evident through the observation of heightened transients subsequent to the peak uncertainty period. Similarly, in the scenario where the model incorporates a considerable count of previous time steps or data points set at 100 for predictions at subsequent time steps, the associated uncertainty estimations are illustrated in Fig. 5f–j. Examination of Fig. 5 yields insights into the impact of expanded past data on uncertainty profiles. With an elevation in the number of preceding data points, a discernible reduction is discerned in the upper bounds of uncertainty estimates for the attention LSTM model, decreasing from $6.75\times 10^{-9}$ to $6.2\times 10^{-9}$. In contrast, the simple LSTM model exhibits a modest increment in uncertainty estimations. This outcome is ascribed to the model’s limited capacity to manage substantial data volumes effectively. Furthermore, a noteworthy shift in the peak of uncertainty is observed in a rightward direction, signaling tempered confidence in the predictive aptitude of the model. Conversely, the GRU model remains predominantly unaffected by the augmentation in historical data points. Concurrently, the residual LSTM model upholds its narrower characteristic uncertainty attributes while exhibiting a marginal reduction in its upper boundary. This adjustment underscores the model’s robust approach to forecasting polarization currents, preserving its reliability under evolving conditions.

**Table 2:** Mean and standard deviation of residuals for different forecasting models in Case 1 and Case 2.

Model	(a) Case 1		(b) Case 2	
	Mean residual	Std dev residual	Mean residual	Std dev residual
Attention LSTM	$-1.6700 \times 10^{-11}$	$5.1807 \times 10^{-11}$	$-1.4836 \times 10^{-11}$	$4.8007 \times 10^{-11}$
LSTM	$-2.9950 \times 10^{-11}$	$7.0636 \times 10^{-11}$	$-2.5608 \times 10^{-11}$	$6.8006 \times 10^{-11}$
GRU	$-1.3892 \times 10^{-11}$	$4.5987 \times 10^{-11}$	$-1.1619 \times 10^{-11}$	$4.2006 \times 10^{-11}$
CNN	$-1.1850 \times 10^{-11}$	$3.2181 \times 10^{-11}$	$-1.1475 \times 10^{-11}$	$3.7358 \times 10^{-11}$
Residual LSTM	$1.2017 \times 10^{-11}$	$2.7876 \times 10^{-11}$	$1.1260 \times 10^{-11}$	$2.3006 \times 10^{-11}$

Table 2a presents an analysis of the residuals obtained from various forecasting models. Residuals signify the discrepancies between the predicted values and actual outcomes in a forecasting context. The Table showcases five distinct forecasting models, including attention LSTM, LSTM, GRU, CNN, and residual LSTM networks. Each model’s performance is evaluated based on mean residual and standard deviation of residual, which are pivotal indicators of the accuracy and consistency of the forecasting models. Mean residual quantifies the average difference between the forecasted values and the actual outcomes. Negative values indicate an overestimation trend, where the forecasted values tend to be slightly higher than the actual outcomes. Conversely, positive values reflect an underestimation trend, suggesting the forecasted values are slightly lower than the actual outcomes. In this investigation, the attention LSTM, LSTM, GRU, and CNN-based forecasting models exhibit a negative mean residual, demonstrating a tendency to overestimate the actual values. However, the proposed residual LSTM model demonstrates a positive mean residual, implying a propensity to underestimate the actual values. Furthermore, the standard deviation of residual measures the dispersion or variability of the forecast errors of models around their mean. Smaller standard deviations indicate consistent and precise predictions, while larger values suggest more variability and potential inconsistencies. Across all models, the standard deviations of residuals are notably small, signifying that the forecasted values remain consistently relative to the actual outcomes. This consistency aligns with the overall accuracy and reliability of the forecasting models. The presence of a positive mean residual in the Residual LSTM model indicates a distinctive predisposition towards underestimation. This characteristic serves to distinguish the Residual LSTM model from the other models, all of which manifest negative mean residuals. Similar results were observed for Case 2 when employing a larger number of historical data points for the forecasting process. Notably, a slightly pronounced enhancement in forecasting outcomes is evident from the data presented in Table 2b.

The conductivity parameter $\sigma _{r}$ can be approximately estimated by obtaining the steady-state DC component value of the forecasted polarization current ($i_{dc}^{pol~for}$) given as follows^14^:

15 $$\begin{aligned} \sigma _{r} \approx \varepsilon _{0} \frac{i_{dc}^{pol~for} }{C_{0} E_{0}} \end{aligned}$$

Table 3 presents a comprehensive overview of the results obtained from investigating the DC conductivity of oil-paper insulation samples using various deep-learning forecasting models. It diverges into two distinct cases, each accompanied by its corresponding parameters and outcomes. The Table presents the forecasted signal, DC conductivity values, and the associated percentage errors associated with each model’s predictions, which have been scaled by a factor of $10^{2}$ for clarity. Notably, it is evident that the residual LSTM model exhibits the lowest percentage error, indicating its superior forecasting accuracy in comparison to the other models across both cases. It compares these deep-learning models’ comparative performance in estimating conductivity for oil-paper insulation samples.

**Table 3:** Parameter estimation from forecasted polarization current.

Forecasting model	Case 1		Case 2	
	$\sigma _{r}$ (DC conductivity)	% Error (scaled by $10^{2}$)	$\sigma _{r}$ (DC conductivity)	% Error (scaled by $10^{2}$)
Attention LSTM	1.7426e−11	6.3	1.7425e−11	5.7
LSTM	1.7424e−11	5.1	1.7422e−11	4.0
GRU	1.7423e−11	4.5	1.7421e−11	3.4
CNN	1.7421e−11	3.4	1.7419e−11	2.3
Residual LSTM	1.7419e−11	2.2	1.7417e−11	1.1

## Discussion

In the systematic assessment of transformer insulation conditions by predicting short-duration polarization currents, a thorough assessment of various predictive models was conducted. A comprehensive evaluation of distinct predictive models was undertaken to develop a short-duration PDC measurement approach, encompassing Attention, LSTM, GRU, XGBoost, CNN, and Residual LSTM network configurations. Notably, the residual LSTM network model showcased a substantial reduction in MAE when contrasted with the baseline XGBoost model for case 2. These findings highlight the efficacy of the proposed forecasting model in capturing intricate temporal relationships within polarization current data, emphasizing its potential to advance predictive accuracy across various predictive horizons. This study investigates the refinement of predictive modeling techniques in analyzing polarization phenomena of transformer insulation, which can be applied to reduce the cumulative measurement time of the time domain dielectric response technique.

Furthermore, the Monte Carlo dropout prediction for uncertainty estimation in polarization current forecasting is implemented for quantifying the uncertainty handling capability of the proposed forecasting model. Multiple passes through deep learning models collect predictions, with mean and standard deviation indicating uncertainty. Analysis reveals that for case 1, the residual LSTM model with the least uncertainty has the lowest peak density, around $0.4 \times 10^{-8}$. Additionally, the simple LSTM model shows the highest peak density of $8\times 10^{9}$, while for attention LSTM model, uncertainty spread lies between $6.2 \times 10^{-9}$ to $6.75 \times 10^{-9}$ with high density. Furthermore, the CNN model depicted comparable performance with the proposed residual LSTM model with a slightly broader spread, indicating higher variability of probability density estimates.

With improved past data, attention to LSTM’s uncertainty decreases, while the simple LSTM’s uncertainty estimates show an increase that limits its capacity due to increases in computational complexity. The performance of the GRU model is still relatively stable; residual LSTM slightly reduces the upper boundary. Within the framework of this investigation, the assessed forecasting models, namely attention LSTM, LSTM, and GRU, collectively display a distinct pattern of negative mean residuals. This pattern implies a consistent inclination to overestimate the actual values during the intended prediction process. In contrast, the proposed residual LSTM model presents an intriguing departure from this trend, exhibiting a positive mean residual. This distinctive characteristic suggests a predisposition of this model to exhibit underestimation tendencies when forecasting actual values. The nuanced variations in mean residuals across these models offer valuable insights for refining various applications of polarization current forecasting scenarios. Furthermore, the insulation-sensitive conductivity parameter derived from the proposed forecasted residual LSTM model shows the least percentage error in both cases. Moreover, a subtle improvement in the conductivity parameter is observed under case 2, which uses more past observed data samples.

## Conclusion

A short-duration polarization current forecasting methodology is proposed in this investigation. The proposed deep-learning approach for short-term polarization current forecasting methodology substantially reduces the cumulative PDC measurement time and its subsequent alteration caused by external disruptions. The experimental results using laboratory-prepared insulation samples demonstrate the proposed residual LSTM model’s efficacy in accurately forecasting polarization current data for a forecast horizon of 1300 s under both cases. Furthermore, the finding suggests that the proposed residual LSTM polarization current forecasting method shows the least MSE, MAE, and RMSE values for case 2. Additionally, the statistical examination of the forecasting model reveals minimal deviation compared to other proposed models, with a mean residual value of $1.1260 \times 10^{-11}$ and a standard deviation of residuals at $2.3006 \times 10^{-11}$ for case 2. Moreover, a Monte Carlo dropout prediction method is utilized to measure the uncertainty of the polarization current forecasting models. Monte Carlo samples signify uncertainty, revealing prediction variability and confidence. Expanded preceding data reduces upper uncertainty bounds in the residual LSTM model. This marginal reduction in its upper boundary exhibits the model’s robust approach to forecasting polarization currents, preserving its reliability under varied conditions. Furthermore, the evaluation of an essential insulation-sensitive parameter $\sigma _{r}$ is conducted using the anticipated polarization current. The findings underscore that the suggested polarization current forecasting methodology can effectively curtail measurement duration by accurately computing insulation-sensitive parameters. This, in turn, prevents external distortions in polarization depolarization current measurements stemming from prolonged measurement periods by reducing the cumulative measurement time.

The polarization current measurements of samples in laboratory conditions exhibited minimal noise. However, it’s noteworthy that in situ measurements could be susceptible to low-frequency noise caused by slow-varying environmental conditions. Future research endeavors could investigate forecasting noise-affected polarization current with a more detailed analysis of multiple insulation-sensitive parameters. This extension of the methodology could offer insights into addressing challenges posed by real-world environmental variations and further enhance the robustness of insulation modeling.

## Data Availability

Please contact corresponding author for data requests.
